# Common *ERBB2 *polymorphisms and risk of breast cancer in a white British population: a case–control study

**DOI:** 10.1186/bcr982

**Published:** 2005-01-12

**Authors:** Patrick R Benusiglio, Fabienne Lesueur, Craig Luccarini, Donald M Conroy, Mitul Shah, Douglas F Easton, Nick E Day, Alison M Dunning, Paul D Pharoah, Bruce AJ Ponder

**Affiliations:** 1 Department of Oncology, University of Cambridge, Strangeways Research Laboratories, Cambridge, UK; 2 Department of Genetic Epidemiology, University of Cambridge, Strangeways Research Laboratories, Cambridge, UK; 3 EPIC, University of Cambridge, Strangeways Research Laboratories, Cambridge, UK

**Keywords:** breast cancer, case–control study, *ERBB2*, haplotype, single-nucleotide polymorphism

## Abstract

**Introduction:**

About two-thirds of the excess familial risk associated with breast cancer is still unaccounted for and may be explained by multiple weakly predisposing alleles. A gene thought to be involved in low-level predisposition to the disease is *ERBB2 (HER2)*. This gene is involved in cell division, differentiation, and apoptosis and is frequently amplified in breast tumours. Its amplification correlates with poor prognosis. Moreover, the coding polymorphism I655V has previously been associated with an increased risk of breast cancer.

**Methods:**

We aimed to determine if common polymorphisms (frequency ≥ 5%) in *ERBB2 *were associated with breast cancer risk in a white British population. Five single-nucleotide polymorphisms (SNPs) were selected for study: SNP 1 near the promoter, SNP 2 in intron 1, SNP 3 in intron 4, SNP 4 in exon 17 (I655V), and SNP 5 in exon 27 (A1170P). We tested their association with breast cancer in a large case–control study (*n *= 2192 cases and 2257 controls).

**Results:**

There were no differences in genotype frequencies between cases and controls for any of the SNPs examined. To investigate the possibility that a common polymorphism not included in our study might be involved in breast cancer predisposition, we also constructed multilocus haplotypes. Our set of SNPs generated all existing (*n *= 6) common haplotypes and no differences were seen in haplotype frequencies between cases and controls (*P *= 0.44).

**Conclusions:**

In our population, common *ERBB2 *polymorphisms are not involved in predisposition to breast cancer.

## Introduction

Breast cancer is the most common cause of cancer in women in the United Kingdom and is, after lung cancer, the most common cause of cancer death (Office for National Statistics). Positive family history is a well-established risk factor for the disease: the risk to first-degree relatives of a breast cancer case is about twice the population risk [1]. Most of the excess familial risk associated with breast cancer is likely to be genetic in origin [2,3]. However, only about a third of this risk is accounted for by known genes, the most important being *BRCA1 *and *BRCA2*, while the remainder might be explained by a combination of weakly predisposing alleles [2-4]. A gene thought to be involved in low-level susceptibility to breast cancer is *ERBB2 *(*HER2*). This gene is located on chromosome 17q12–q21, spans 38 kilobases, and comprises 27 coding exons. It is a member of the ERBB family, a family of protein tyrosine kinases involved in cell division, migration, adhesion, differentiation, and apoptosis and consisting of EGFR (ERBB1), ERBB2, ERBB3, and ERBB4 [5]. *ERBB2 *amplification or overexpression is seen in about 25% of breast cancers and has been associated with metastatic phenotype, endocrine therapy unresponsiveness, and poor prognosis [6]. *ERBB2 *is polymorphic in the transmembrane region of the protein at codon 655 (ATC/isoleucine to GTC/valine [I655V]). The amino acid change could result in increased protein tyrosine kinase activity [7]. Several association studies of I655V and breast cancer risk have yielded conflicting results. In a study on 700 Han Chinese women, Xie and colleagues first reported a significantly increased risk for carriers of the rare allele (odds ratio [OR] = 1.4) [8]. Only one of seven subsequent studies showed an overall effect of I655V on breast cancer risk [9-15]. However, of the negative studies, all but one had limited power to detect a risk of this magnitude [13]. Three groups did report associations in specific subgroup analyses in the absence of overall effect: Wang-Gohrke and Chang-Claude showed an association in women with a positive family history of breast cancer and McKean-Cowdin and colleagues showed an association with localized breast cancer, whereas Millikan and colleagues showed an association in women with a positive family history who were aged 45 years or younger as well as an increased risk of carcinoma *in situ *[12,13,15].

I655V has usually been selected for study because of the possible functional consequences of the amino acid change in the transmembrane region of the protein. Many more single-nucleotide polymorphisms (SNPs) in *ERBB2 *are known but only one negative study has reported on any of these [10]. A selected set of sequence polymorphisms can serve as genetic markers to detect association between a particular region and the disease, whether or not the markers themselves have a functional effect [16]. It is therefore not necessary to test each polymorphism individually. Because most SNPs are correlated with nearby polymorphisms, genotypes at unsassayed, risk-related SNPs will be correlated with one or more assayed SNPs [17]. If the set of selected markers provides enough information about the remainder of the common polymorphisms in that gene, any susceptibility allele within or close to the gene should be uncovered through the evaluation of the underlying haplotypes [18]. To clarify the role of *ERBB2 *in the predisposition to breast cancer, we tested the association of five common polymorphisms (including I655V) with the disease in a large case–control study of white British women. We aimed to identify sufficient SNPs to tag all the common haplotypes across the gene.

## Materials and methods

### Patients and controls

Cases were drawn from the Anglian Breast Cancer Study, an ongoing population-based study with cases ascertained through the East Anglian Cancer Registry [4]. All women diagnosed with invasive breast cancer under the age of 55 years between 1 January 1991 and 30 June 1996 and who were alive at the start of the study (prevalent cases) as well as women under the age of 70 who were diagnosed from 1996 onwards (incident cases) were eligible for inclusion. We used prevalent and incident cases in order to maximize sample size; approximately 65% of eligible patients have enrolled in the study. Women taking part in the study were asked to provide a 20-ml blood sample for DNA analysis and to complete a comprehensive epidemiological questionnaire. We carried out genotyping on a subset consisting of the first 2192 (1438 incident and 754 prevalent) enrolled cases. Controls (2257) were randomly drawn from the Norfolk component of the European Prospective Investigation of Cancer (EPIC) [19]. The ethnic background of both cases and controls is similar, with over 98% being white Anglo-Saxon. Ethical approval was obtained from the Anglia and Oxford Multicentre Research Committee and informed consent was obtained from each patient.

### SNP identification and selection

SNPs with validated frequency data were identified in January 2004 through the dbSNP database . If these data were from a non-Caucasian population, we confirmed the presence of the polymorphism in our population by performing denaturing high-performance liquid chromatography on a set of 48 genomic DNA samples from UK breast cancer patients. We selected all nonsynonymous coding SNPs (*n *= 2), SNPs located in the promoter region (*n *= 1), and two randomly chosen intronic SNPs [20]. A total of five SNPs were thus selected for study (Table 1). In order to have good power to detect small relative risks, we restricted our attention to SNPs with a frequency of 5% or more.

### Genotyping

Genotyping was carried out using Taqman^® ^(Applied Biosystems, Warrington, UK) according to the manufacturer's instructions. Primers and probes were either supplied directly by Applied Biosystems in case of Assays-by-Design™ (SNP 1 and SNP 2) and Assays-on-Demand™ (SNP 3) or designed using Primer Express Oligo Design Software v2.0 (Applied Biosystems) (SNP 4 and SNP 5). Sequences are available on request. Reactions were carried out at 54°C (SNP 4) or 60°C (SNP 1, SNP 2, SNP 3, and SNP 5). All assays were carried out in 384-well plates. Each plate contained 384 samples including 2 negative controls with no DNA and 12 samples duplicated on a separate quality-control plate. Plates were read on the ABI Prism 7900 using the Sequence Detection Software (Applied Biosystems). Failed genotypes were not repeated.

### Statistical methods

The characteristics of cases and controls were explored with SPSS^© ^v12.0.1 (SPSS Inc, Chicago, IL, USA). For each SNP, deviation of genotype frequencies in controls from the Hardy–Weinberg equilibrium was assessed by χ^2 ^test with one degree of freedom (df). Genotype frequencies in cases and controls or within cases stratified by disease stage (stage I vs stages II–IV) or age group (≤ 45 vs >45) were compared by χ^2 ^test for heterogeneity (2df). Genotype-specific risks were estimated as ORs using standard cross-product ratio. Confidence intervals were calculated using the variance of the log (OR), which was estimated by the standard Taylor expansion. Power was determined using standard statistical methods [21]. We have over 90% power at the 1% significance level to detect a dominant allele with a frequency of 0.05, which confers a relative risk of 1.5, or a dominant allele with a frequency of 0.2 that confers a relative risk of 1.3. Power to detect recessive alleles at the 1% significance level is more limited: 59% for an allele with a frequency of 0.2 that confers a relative risk of 1.5 or 77% for an allele with a frequency of 0.3 that confers a relative risk of 1.4. The LDA program [22] was used to calculate pairwise linkage disequilibrium (LD) for each SNP pair in the whole case–control set. The haplo.score program [23] was used to test for association between haplotypes and breast cancer risk. Haplo.score uses a likelihood that depends on estimated haplotype frequencies to test the statistical association between haplotypes and phenotype. It is based on score statistics, which provide both global tests and haplotype-specific tests [23].

## Results

**Figure 1:**
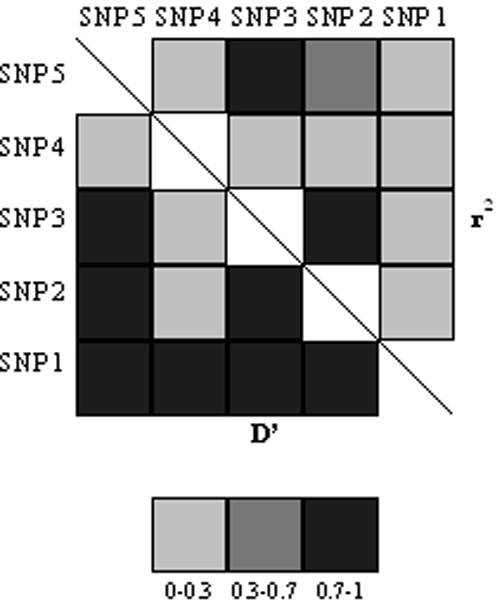
Pairwise linkage disequilibrium (LD) measures of D' (left bottom half) and *r*^2 ^(right top half) for the five SNPs.

The median age was 48 years (range 25–54) for prevalent cases, 52 years (26–55) for incident cases, and 56 years (25–81) for controls. Incident and prevalent cases were similar regarding breast cancer stage (*P *= 0.12) and histological grade (*P *= 0.41). Table 2 shows the genotype frequencies in cases and controls as well as genotype-specific risks for the five SNPs assayed. The genotype frequencies were similar in the prevalent and incident cases for all polymorphisms (data not shown). None of the genotype distributions for the controls differed significantly from those expected under Hardy–Weinberg equilibrium. There was no evidence that any of the SNPs is associated with breast cancer; genotype-specific ORs were all close to unity with narrow confidence intervals. We also compared genotype frequencies within cases stratified by disease stage and age group for SNP 4 (I655V). No differences were seen (*P *[stage] = 0.61, *P *[age group] = 0.33). LD was strong (D' > 0.7) across pairs involving SNPs 1, 2, 3, and 5, whereas SNP 4 was in weak LD (D' < 0.3) with all other polymorphisms except SNP 1 (D' [SNP 1-SNP 4] = 0.98) (Fig. 1). SNPs 3 and 5 were in nearly perfect LD (*r*^2 ^= 0.92). Of 32 possible haplotypes, only 6 were observed with a frequency greater than 5% (Table 3). For the whole case–control set, common haplotypes constituted 98% of all the observed haplotypes. Two haplotypes (haplotypes 3 and 5) contained the SNP 4 (I655V) minor allele. The global test was not significant (*P *= 0.44), nor were there any differences between cases and controls for individual haplotypes. Similarly, no differences in haplotype frequencies were seen within cases stratified by disease stage (*P *= 0.37) or age group (*P *= 0.48).

## Discussion

Our study is the largest case–control study reported on *ERBB2 *genetic variation. To our knowledge, this is also the first study on *ERBB2 *reporting results for more than two polymorphisms and looking for involvement of haplotypes in breast cancer predisposition. We performed a study of five common SNPs and found no evidence for association with breast cancer risk. Four of the polymorphisms may be functional: SNP 1 near the promoter region and SNP 2 in intron 1 could be involved in regulatory processes whereas SNP 4 and SNP 5 are nonsynonymous coding SNPs that could affect tyrosine kinase activity or protein structure [7]. Two association studies have previously reported a positive association between SNP 4 (I655V) and breast cancer risk [8,14]. Both genotyped about 700 individuals and showed a similarly increased risk for carriers of the Val allele (OR = 1.4). We were not able to replicate these findings. We have over 90% power to detect a risk of this magnitude at the 10^-4 ^level of significance. This suggests that previous positive findings may have been due to type I statistical errors. Neither could we replicate findings associating I655V with low-stage breast cancer or with breast cancer in younger women [12,13]. Positive results from stratified analyses should be treated with caution; very large sample sizes are required to obtain reliable results, the number of possible analyses that can be undertaken is large, and there is a strong possibility that one or more tests will be statistically significant simply by chance [24]. We could not carry out analyses within cases stratified by family history, because we only had incomplete family history data [15]. To investigate the possibility that a common polymorphism not included in our study might be involved in breast cancer predisposition, we constructed multilocus haplotypes and observed similar frequencies in cases and controls. We found six common haplotypes. Recently, the NIEHS Environmental Genome Project at the University of Washington released resequencing data based on 90 individuals (the PDR90 population; individual genotypes are available on line: ) and identified nine common SNPs (frequency ≥ 5%) in *ERBB2*. All the common haplotypes (frequency ≥ 5%) were tagged by our set of five SNPs, even though, as expected given the multiethnicity of PDR90, differences in frequencies were seen between the two populations (data not shown). Crawford and colleagues resequenced 100 candidate genes involved in inflammation, lipid metabolism, and blood pressure regulation and showed that in a population of European descent the average number of common haplotypes per gene was 4.5, with a maximum number of 8 observed in only two genes [25]. We are therefore confident that we have detected all common *ERBB2 *haplotypes present in our population. We limited our study to common polymorphisms. A larger study set would be needed to identify a rarer polymorphism involved in disease predisposition. For example, dominant alleles with a frequency of 2% would require more than 4000 cases and 4000 controls to detect a relative risk of 1.5 significant at the 1% level with 90% power. We cannot exclude the possibility that a common SNP might have a differential effect in another ethnic group via gene–gene or gene–environment interactions, or that a predisposing SNP might be present exclusively in another population [26]. In summary, we conducted a large case–control study of *ERBB2 *and breast cancer. We genotyped five common SNPs, including the much-studied I655V polymorphism, and saw no association with the disease. Our set of SNPs generated all common haplotypes, and no differences in haplotype frequencies were seen between cases and controls.

## Conclusion

In our population, common *ERBB2 *polymorphisms are not involved in predisposition to breast cancer.

## Abbreviations

df = degree of freedom; LD = linkage disequilibrium.; OR = odds ratio; SNP = single-nucleotide polymorphism.

## Competing interests

The author(s) declare that they have no competing interests.

## Authors' contributions

PRB performed the experiments, carried out the analyses, and wrote the manuscript under the supervision of FL, PDP, and BAJP. CL and DMC managed the genotyping process. MS and NED supervised DNA samples collection. DFE was the statistical advisor and AMD was the laboratory manager. All authors read and approved the final manuscript.

## Figures and Tables

**Table 1:** Single-nucleotide polymorphisms (SNPs) selected for genotyping

SNP name	dbSNP id	Location	Base change
SNP 1	rs4252596	START -657^a^	C>A^b^
SNP 2	rs2952155	intron 1	C>T
SNP 3	rs1810132	intron 4	T>C
SNP 4	rs1801200	exon 17 (I655V)	A>G
SNP 5	rs1058808	exon 27 (A1170P)	G>C

^a^657 base pairs upstream first translated base. ^b^Common allele given first.

**Table 2:** Genotype frequencies and genotype-specific risks in 2192 women with breast cancer and 2257 controls

SNP	Series	Rare-allele frequency	Common homozygote No. (%)	Heterozygote No. (%)	Rare homozygote No. (%)	Number genotyped	*P *(2df)
SNP 1	Cases	0.13	1618 (77)	447 (21)	42 (2)	2107	
	Controls	0.13	1645 (75)	511 (23)	33 (2)	2189	0.13
	OR (95% CI)		1 (ref)	0.89 (0.77–1.03)	1.29 (0.82–2.05)		
SNP 2	Cases	0.25	1151 (57)	734 (36)	140 (7)	2025	
	Controls	0.26	1219 (55)	839 (38)	147 (7)	2205	0.48
	OR (95% CI)		1 (ref)	0.93 (0.82–1.05)	1.01 (0.79–1.29)		
SNP 3	Cases	0.32	962 (47)	855 (42)	219 (11)	2036	
	Controls	0.32	1022 (46)	956 (43)	230 (11)	2208	0.69
	OR (95% CI)		1 (ref)	0.95 (0.84–1.08)	1.01 (0.82–1.24)		
SNP 4	Cases	0.25	1128 (57)	748 (37)	113 (6)	1989	
	Controls	0.25	1230 (57)	791 (37)	134 (6)	2155	0.69
	OR (95% CI)		1 (ref)	1.03 (0.91–1.17)	0.92 (0.71–1.20)		
SNP 5	Cases	0.33	911 (45)	870 (43)	233 (12)	2014	
	Controls	0.33	960 (44)	983 (45)	238 (11)	2181	0.45
	OR (95% CI)		1 (ref)	0.93 (0.82–1.06)	1.03 (0.84–1.26)		

OR, odds ratio; SNP, single-nucleotide polymorphism.

**Table 3:** Estimated haplotype frequencies in cases and controls

Haplotype	Allele^a^	Frequency (cases)	Frequency (controls)	*P*^b^
(1)	cctIA	0.35	0.36	0.80
(2)	ctcIP	0.19	0.18	0.88
(3)	cctVA	0.18	0.16	0.15
(4)	actIA	0.13	0.13	0.49
(5)	ctcVP	0.06	0.08	0.07
(6)	cccIP	0.07	0.07	0.50
(7)	cctIP	0.02	0.01	0.93

^a^Polymorphisms in 5' to 3' order as indicated in Table 1. ^b^Global score statistic = 6.91, df = 7, *P *= 0.44
